# Formulation and Characterization of Ursodeoxycholic Acid Nanosuspension Based on Bottom-Up Technology and Box–Behnken Design Optimization

**DOI:** 10.3390/pharmaceutics15082037

**Published:** 2023-07-28

**Authors:** Oriana Boscolo, Sabrina Flor, Leandro Salvo, Cecilia Dobrecky, Christian Höcht, Valeria Tripodi, Marcela Moretton, Silvia Lucangioli

**Affiliations:** 1 Universidad de Buenos Aires, Facultad de Farmacia y Bioquímica, Departamento de Tecnología Farmacéutica, Buenos Aires C1113AAD, Argentina; oboscolo@ffyb.uba.ar (O.B.); sflor@ffyb.uba.ar (S.F.); lsalvo@ffyb.uba.ar (L.S.); lcdobrec@ffyb.uba.ar (C.D.); mmoretton@ffyb.uba.ar (M.M.); 2 Universidad de Buenos Aires, Facultad de Farmacia y Bioquímica, Instituto de Tecnología Farmacéutica y Biofarmacia (InTecFyB), Buenos Aires C1113AAD, Argentina; chocht@ffyb.uba.ar (C.H.); vtripodi@ffyb.uba.ar (V.T.); 3 Consejo Nacional de Investigaciones Científicas y Técnicas (CONICET), Buenos Aires C1425FQB, Argentina; 4 Universidad de Buenos Aires, Facultad de Farmacia y Bioquímica, Departamento de Farmacología, Buenos Aires C1113AAD, Argentina; 5 Universidad de Buenos Aires, Facultad de Farmacia y Bioquímica, Departamento de Ciencias Químicas, Buenos Aires C1113AAD, Argentina

**Keywords:** nanosuspension, ursodeoxycholic acid, Box–Behnken design, bottom-up technology

## Abstract

Background: Ursodeoxycholic acid (UDCA) is a therapeutic agent used for the treatment of cholestatic hepatobiliary diseases in pediatric patients. It is a bile acid that presents high lipophilicity, and it belongs to Class II of the Biopharmaceutical Classification System (BCS), which exhibits low water solubility and high intestinal permeability, which leads to poor oral absorption. The objective of this work was to design and optimize UDCA nanosuspensions by means of the precipitation-ultrasonication method to improve the solubility, dissolution, and oral bioavailability of UDCA. Methods: A three-level, three-factor Box–Behnken design was used to optimize formulation variables and obtain uniform, small-particle-size UDCA nanosuspensions. The independent variables were: stabilizer percentage (*X*_1_), amplitude (*X*_2_), and sonication time (*X*_3_), and the dependent variable was the particle size (*Y*_1_). In the precipitation–ultrasonication method, UDCA was dissolved in acetone:PEG 400 (1:1 *v*/*v*) and quickly incorporated into the antisolvent (pre-cooled aqueous dispersion of HPMC E-15 0.3%), by means of intense sonication at 50 W for 5 min, controlling temperature through an ice water bath. The lyophilization efficacy was evaluated by means of a cryoprotective efficacy test, working with 10% maltose at −80 °C. The nanosuspensions were characterized by dynamic light scattering (DLS), X-ray diffraction, and scanning electron microscopy (SEM). The physicochemical stability was determined at 25 °C and 4 °C at 7, 14, 30, and 60 days, and the UDCA content was analyzed via HPLC-UV. An in vitro dissolution assay and an oral bioavailability study were performed in male Wistar rats. Results: A significant impact was achieved in the optimized nanosuspension with 0.3% (stabilizer), 50 W (amplitude), and 5 min (sonication time), with a particle size of 352.4 nm, PDI of 0.11, and zeta potential of −4.30 mV. It presented adequate physicochemical stability throughout the study and the UDCA content was between 90% and 110%. In total, 86% of UDCA was dissolved in the in vitro dissolution test. The relative oral bioavailability was similar without significant statistical differences when comparing the lyophilized nanosuspension and the commercial tablet, the latter presenting a more erratic behavior. The pharmacokinetic parameters of the nanosuspension and the commercial tablet were T_max_ (1.0 ± 0.9 h vs. 2.0 ± 0.8 h, respectively), C_max_ (0.558 ± 0.118 vs. 0.366 ± 0.113 µM, respectively), ΔC_max_ (0.309 ± 0.099 vs. 0.232 ± 0.056, respectively), AUC (4.326 ± 0.471 vs. 2.188 ± 0.353 µg/mL.h, respectively, *p* < 0.02), and IAUC_0–24h_ (2.261 ± 0.187 µg/mL.h vs. 1.924 ± 0.440 µg/mL.h, respectively). Conclusions: The developed nanosuspension presents an appropriate dosage and administration for pediatric patients. On the other hand, it exhibits an adequate absorption and UDCA oral bioavailability.

## 1. Introduction

In recent years, the development of a formulation with optimal bioavailability for those hydrophobic active pharmaceutical ingredients (APIs) with poor aqueous solubility that belong to class II or IV (biopharmaceutical classification system or BCS) has been a great challenge for pharmaceutical scientists. This poor aqueous solubility and dissolution rate lead to erratic absorption and poor oral bioavailability [1]. Many strategies have been developed to overcome this problem, such as nanosuspension technology. It is based on a dispersion of pure drug nanocrystals (100–1000 nm) in water with a minimum number of surface-active agents (stabilizing polymers and/or surfactants) necessary for stabilization [2]. These systems present a series of advantages with respect to other techniques, such as improved bioavailability due to an increase in surface area, dissolution rate, and oral absorption as a consequence of the reduction in particle size; easy preparation; lower toxicity and side effects; long-term physical stability; sustained and controlled release effects; reduced excipient use; and decreased dosing frequency which leads to increased patient compliance [2,3,4,5,6]. Nanosuspension particle size limit is less than 1 µm with an average range between 200 and 600 nm [5]. This is achieved through various techniques, such as “Bottom-up”, “Top-down”, or the combination of both [7]. “Top down” techniques or disintegration methods involve mechanical crushing processes for the conversion of large particles into fine particles by using high-pressure homogenization (HPH) or media milling. Although these methods are more feasible from an industrial perspective, they are expensive as long processing times are required, and they present contamination problems due to residual metals coming from the equipment. In turn, as high-energy processes are involved, caution should be taken with heat-sensitive drugs [2,4,8,9]. The “bottom up” techniques or precipitation methods start from the molecular level, and through molecular associations the formation of solid particles is reached [10]. This method involves the precipitation in crystalline or amorphous form from a supersaturated solution by addition of an antisolvent or by evaporation of the solvent. In the antisolvent precipitation method, the drug is first dissolved in a water-soluble organic solvent and then rapidly mixed with the antisolvent (pre-cooled aqueous solution containing stabilizers) by stirring. Mixing the drug, previously dissolved in a solvent, with the antisolvent results in a higher supersaturation, and this leads to the formation of small particles [11,12]. The properties of the coprecipitated particles depend on the formulation variables (drug and polymer characteristics, solvent/antisolvent ratio, drug–polymer interaction) and the operating parameters (temperature, stirring speed, mixing time) [13]. In recent years, numerous nanosuspensions have been developed using the antisolvent technique [14,15,16]. The “bottom up” technique allows the formation of finely dispersed small and uniform drug particles, and their size and distribution may be modified by changing the operating parameters [17]. The advantages of this technique are the use of simple and low-cost instruments, low energy requirements, and easy scaling up. On the other hand, one limitation of this method is related to the need for residual solvent elimination for safety purposes when an organic solvent is used in the nanosuspension development process. In addition, by-product impurities that may appear in the precipitation process and the difficulty of adequately controlling the final size of the crystal are other concerns. [6,8,11,13,18]. Hence, in order to obtain an improved product, ultrasonication was introduced as an effective method, which has been combined with precipitation to control nucleation processes and crystal growth using confined jet mixers or multi-entry vortex mixers (flash nanoprecipitation) [19,20,21,22]. When ultrasonication is applied to liquids, ultrasound waves are characterized by a cyclical succession of expansion and compression phases. In the compression phase, the molecules of the liquid are pushed, and in the expansion phase, the molecules are separated. In turn, the ultrasound waves intensify the mass transfer by initiating cavitation. In the compression phase, bubbles are formed. Cavitational forces and the phenomena of microbubble formation, growth, and collapse release a large amount of energy. When a bubble collapses, it forms a confined hot spot with high temperature and pressure, which releases shock waves. In this way, the mixing of the solvent and the anti-solvent is improved, and supersaturation of the mixture occurs. Furthermore, the implosion of the vacuum bubbles breaks the particles apart. The results of this process depend on the duration and intensity of sonication, the length of the tube and the depth of immersion, and the temperature [23].

To obtain a UDCA nanosuspension based on a bottom-up technique, the identification of formulation and process variables that influence the size and the polydispersion index was the main objective of our work. Traditionally, the optimization method involved “changing one variable at a time (OVAT), while holding others constant”, which is a laborious, expensive, and time-consuming process. Therefore, quality by design emerged as an alternative, efficient, and cheap method that not only studies each variable individually, but also their interaction and can provide a mathematical model to explain and predict these relationships. RSM (response surface method) with Box–Behnken design (BBD) was employed to determine this optimal condition. BBD is a rotatable second-order design used to generate higher-order response surfaces using fewer required runs than a normal factorial technique. BBD has been extensively used to study the variables involved in formulation processes. In a BBD design, the factors are the independent variables, while responses correspond to the dependent variables. Each factor is analyzed at three levels. A three-factor and three-level (3^3^) BBD reduces the number of experiments and statistically optimizes the variables of the nanosuspension formulation in order to obtain a small and uniform particle size [2,24,25,26,27,28,29].

Ursodeoxycholic acid (UDCA) (3a,7b-dihydroxy-5b-cholan-24-oic acid), or ursodiol, is a secondary bile acid that is currently approved as a therapeutic agent for hepatobiliary disorders as it improves the histological features and restores hepatic function and biochemical parameters in children with different cholestatic diseases. It is a cholagogue, liver protector, and cholelitholytic agent, which presents a low bioavailability when orally administered as it belongs to Class II of the Biopharmaceutic Classification System (BCS) due to its poor water solubility in water and high intestinal permeability [30,31]. Different pharmaceutical UDCA dosage forms are available on the market, such as tablets and capsules and an oral liquid formulation, a 250 mg/5 mL suspension (Ursofalk, Biotoscana 2020) [32]. It contains benzoic acid as a preservative. It is important to emphasize that the use of benzyl alcohol and benzoic acid in formulations for pediatric patients (neonates and infants) is discouraged as they have immature metabolizing enzymes, leading to accumulation and ultimately toxicity [33]. UDCA exhibits a low absorption and bioavailability profile. It is orally administered in a non-conjugated form and, since the solubility in the gastrointestinal tract depends on the pKa, this becomes one of the critical factors for its absorption, (UDCA pKa = 5.1). UDCA solubility increases as the pH increases and is optimal at pH higher than 7–8, thus being important to maintain a high pH at the absorption site. Once administered, UDCA is absorbed mainly in the jejunum and ileum, reaching the plasma concentration peak after 30–50 min. Subsequently, it presents a first hepatic pass of 50% [30,34,35].

Nanosuspensions are an attractive alternative to the traditional delivery technologies for drugs that are poorly soluble in water, such as UDCA. In general, nanosuspensions contain stabilizers to prevent instability processes such as caking, agglomeration, and subsequent particle settling and Ostwald ripening [5,36]. Stabilizers adsorption on the drug particle surface generates electrostatically or sterically repulsive barriers that prevent particle aggregation. Different types of stabilizers are used, from polymers such as cellulose derivatives (Hydroxypropyl methylcellulose (HPMC), hydroxypropyl cellulose (HPC), ethylcellulose (EC)), D-α-tocopherol polyethylene glycol 1000 succinate (TPGS), polyvinyl alcohol (PVA), and polyvinylpyrrolidone (PVP); copolymers such as poloxamers (Pluronics) and poloxamines (Tetronic 1107, 148, 407) and ionic surfactants such as phospholipids, sodium dodecyl sulfate (SDS), sodium lauryl sulfate (SLS), and nonionic surfactants (Tween 80) [13,37].

Interestingly, only a few articles of UDCA oral nanosuspensions using high-pressure homogenization (HPH) have been reported [31,38]. Moreover, a combination of high-pressure precipitation tandem homogenization technology (HP-HHT) was also explored to overcome the limitations of traditional technologies for particle size reduction, and it was compared with the conventional HPH method (Li Y. et al.) [39]. Furthermore, other techniques such as nanoprecipitation based on acid–base neutralization by central compound design have been studied to develop amorphous UDCA nanosuspension [40].

The main objectives in order to improve the oral UDCA bioavailability were: (1) to develop and optimize UDCA oral nanosuspensions with the precipitation–ultrasonication method followed by freeze-drying. To this purpose, a BBD was used to optimize variables such as stabilizer concentration, amplitude, and sonication time; (2) to evaluate the physicochemical characteristics of the freeze-dried UDCA nanosuspensions; (3) to study the physicochemical stability at two temperatures; (4) to perform an in vitro dissolution test; and finally (5) to carry out a relative oral bioavailability study of the freeze-dried UDCA nanosuspensions. To the best of our knowledge, no article has been published on the development of UDCA nanosuspensions using bottom-up technology (precipitation–ultrasonication) followed by freeze-drying along with a physicochemical stability and a relative oral bioavailability study.

## 2. Materials and Methods

### 2.1. Materials

UDCA raw material was purchased from Parafarm (Buenos Aires, Argentina); Polyethylene glycol 400 (PEG 400) was provided from Sigma-Aldrich (St. Louis, MO, USA) and Hydroxypropylmethylcellulose E-15 (HPMC) was obtained from Parafarm (Buenos Aires, Argentina); acetone was provided by Sintorgan (Buenos Aires, Argentina); Poloxamine 1107 (Tetronic 1107, T1107, MW 15 kDa) was procured by BASF Corporation (Florham Park, NJ, USA); D-α-tocopherol polyethylene glycol 1000 succinate (TPGS) was obtained from Eastman Chemical Company (Kingsport, TN, USA); maltose was provided by Boehringer Ingelheim (Ingelheim am Rhein, Germany) and mannitol was obtained from J. T. Baker (Philipsburg, NJ, USA).

### 2.2. HPLC Analysis

UDCA concentration was determined by HPLC-UV [41]. HPLC was equipped with a reverse phase C18 column—Waters Symmetry (150 mm × 4.6 mm, id; 5 μm particle size). The mobile phase was acetonitrile-phosphoric acid (pH 3.0, 0.15 mM) (48:52). The flow rate was set to 1 mL/min, with an injection volume of 100 µL, and the oven temperature was set to 40 °C. The detection wavelength was 200 nm.

### 2.3. Experimental Design

A BBD of three factors and three levels was applied to reduce the number of experiments and optimize the variables in the formulation of the UDCA nanosuspensions.

Prior to applying the BBD experimental design, a preliminary examination was carried out to choose the best stabilizer. HPMC E-15, TETRONIC 1107, and TPGS were used to prepare different UDCA nanosuspensions, and the best stabilizer was chosen according to size and particle size distribution, determined by the polydispersity index (PDI).

Stabilizer concentration and processing factors such as time and amplitude were optimized using BBD. To do so, a 3-factor design with 3 levels and 3 repetitions in the central point was used, giving a total of 15 experiments. The independent variables were stabilizer percentage (*X*_1_*)*, amplitude (*X*_2_), and sonication time (*X*_3_). The levels of each factor were designated as (−1, 0 and +1). Particle size (*Y*_1_) was selected as a dependent variable. Table 1 shows the composition of the 15 formulas in standard order. Data evaluation was carried out using an algorithm to obtain the best-fit mathematical model using the Matlab R2017a program.

### 2.4. Preparation of UDCA Nanosuspensions

#### 2.4.1. Precipitation–Ultrasonication Technique

HPMC-stabilized UDCA nanosuspensions were prepared using the bottom-up nanoprecipitation with antisolvent technique, followed by ultrasonication to control the crystal nucleation and growth process [19]. First, 50 mg of UDCA raw material was dissolved in 2 mL of a solvent mixture, acetone: PEG 400 (1:1 *v*/*v*, organic phase). The anti-solvent phase was prepared by dispersing the HPMC E-15 (0.3%; 0.5% and 1.0%) in 40 mL of water with stirring in a Multistirrer 15 shaker (VELP Scientifica, Via Stazione, Usmate (MB), Italy). Then, the anti-solvent phase was sonicated with an ultrasonicator (Qsonica sonicators, Q700; New York, NY, USA) for 3 min. The organic phase was then rapidly incorporated into the 40 mL of the anti-solvent phase by intense sonication at different powers (30 W; 50 W and 100 W) for 5 min. During the process, the temperature was controlled using an ice-water bath. The nanosuspension was subjected to centrifugation (Hanil Science Industrial Co., Combi-514R, Gimpo, Republic of Korea) at 13.500 rpm, at 4 °C, for 1 h. The supernatant was removed and replaced with the same amount of anti-solvent phase. The solid residue was redispersed by ultrasound for 3 min [21].

#### 2.4.2. Particle Size, Distribution, and Zeta Potential of UDCA Nanosuspensions

Hydrodynamic diameter (particle size), size distribution (polidispersity index (PDI)), and zeta potential were determined by dynamic light scattering (DLS) at 25 °C using the Zetasizer Nano-ZSP equipment ZEN 5600 (scattering angle of θ = 173° to the incident beam, Malvern Instruments, Malvern, UK). Samples were diluted 1/10 with ultrapure water and equilibrated for 5 min at 25 °C. The data obtained were analyzed using the Malvern Instruments CONTIN algorithms. The particle size and PDI results were expressed as the average of three measurements ± standard deviation (SD).

#### 2.4.3. Formulation Optimization

The optimized UDCA nanosuspension was obtained using the algorithm developed by Dr. Jorge Magallanes, researcher at CONICET and CNEA (National Atomic Energy Commission, Buenos Aires, Argentina) through the Matlab program. The optimized nanosuspension was prepared and evaluated in triplicate.

#### 2.4.4. Preparation and Characterization of Freeze-Dried UDCA Nanosuspensions

For the stability study, optimal UDCA nanosuspensions were subjected to preliminary cryoprotective efficacy tests. Firstly, nanosuspensions were placed in amber glass vials (5 mL) and they were frozen at two freezing rates (−20 °C and −80 °C) after the addition of either mannitol or maltose as cryoprotectant additives (final cryoprotectant concentration: 10%). Secondly, samples were stored at the selected temperature overnight and freeze-dried for 48 h (Condenser temperature of −80 °C and a pressure of 100 mTorr; Freeze Dryer −90 °C; Operon Co., Ltd., Gyeonggi-do, Republic of Korea). Once lyophilized, the samples were resuspended in the original volume of ultrapure water. Lyophilization efficiency was evaluated by calculating the Sf/Si ratio for every sample (where Sf and Si were the particle size before and after the freeze and thaw cycles, respectively). Those samples providing a Sf/Si ratio of 1 ± 0.3 were selected for further assays [42].

Particle size, PDI, and zeta potential were assessed by DLS at 25 °C using a Zetasizer Nano-ZSP equipment (Malvern Instruments, Malvern, UK), under the same conditions mentioned in Section 2.4.2. The freeze-dried nanosuspension was resuspended in the original ultrapure water volume and manually stirred to obtain a uniform dispersion. Then, a 1/10 dilution was made with ultrapure water. The samples were analyzed in triplicate and the results expressed as the mean ± SD.

Morphological evaluation of UDCA particles in the freeze-dried nanosuspension was performed using scanning electron microscopy (SEM) (FEG-SEM, Zeuss Supra 40 apparatus with a Gemini column, Germany), operated at a 3.0 kV acceleration voltage. The samples were glued and mounted on platinum-coated metal plates at 30 mA for 40 s.

### 2.5. Residual Solvents (Acetone)

#### 2.5.1. Instrumental and Chromatographic Conditions

Gas chromatography equipment coupled to Perkin Elmer model Clarus 500 GC-FID-MS ion detectors with a split/splitless automatic injector (split ratio 1: 100) was used. A silica capillary column of 5% phenyl-95% dimethylpolysiloxane (DB-5, J&W Scientific, Folsom, CA, USA), 60 m × 0.25 mm and 0.25 μm was employed, using helium as carrier gas at 1.87 mL/min. The column was connected to a FID detector and the quadrupole mass detector (70 eV) through a ventilation system (MSVent™, Waltham, MA, USA). The operating conditions were 45 °C (isothermal) for 20 min, injector and mass temperature 150 °C, transfer line temperature 180 °C, and the MS spectra were collected within *m*/*z* 32 to 400. For GC-FID analysis the detector temperature was set at 275 °C. Then, 0.5 µL of sample was injected and the run time was 20 min. The chromatograms were analyzed by TotalChrom 5.1 software.

#### 2.5.2. Stock and Sample Solutions

A methanol: acetone mixture was injected to verify that the resolution of both peaks was adequate (6.15 and 6.75 min, respectively). Standard solutions of acetone in methanol were prepared.

Freeze-dried UDCA nanosuspensions were suspended in methanol and sonicated for 20 min. The samples were run in FULL SCAN and SIM mode.

Linearity, LOD, and LOQ

Linearity was evaluated in three acetone concentration levels (5000, 500, and 50 ppm), where each concentration level was analyzed in duplicate. The regression coefficients were obtained by plotting the average peak area as a function of each concentration, using the least-squares method. Both 0.5 μL and 1 μL of the solutions were injected with an automatic injector and analyzed in full-scan mode.

The LOD and LOQ were determined with a signal/noise ratio (S/R) of 3 and 10, respectively.

### 2.6. Dissolution Test

The dissolution test was carried out according to USP [43]. Not less than 80% of the labeled UDCA content must dissolve in 30 min.

The dissolution medium consisted of 1000 mL of 0.05 M phosphate buffer (pH 8.4) with 2% SLS. Apparatus 2 was used at 37 ± 0.5 °C and 75 rpm. The lyophilizate (corresponding to 5 mL of UDCA nanosuspension) was accurately weighed (~0.2 g) and dispersed in the dissolution medium. The reference sample was a 150 mg UDCA tablet (Urzac, Eurofarma, Argentina) and the procedure was the same as with the freeze-dried sample. Aliquots were taken from the dissolution medium (20 mL) after 30 min and filtered (nylon filter, 45 µm), and then a solid-phase extraction (SPE) was performed (Phenomenex Strata C18-E, 500 mg/3 mL, Torrance, CA, USA). The eluted volume (1 mL) was directly injected into the chromatographic system. The injection volume was 100 µL and the UDCA content was analyzed by HPLC with UV detection. The UDCA standard was diluted in dissolution medium (1/25) followed by SPE. The tests were carried out in triplicate [44].

### 2.7. In Vitro Physicochemical Stability

The stability of the lyophilized UDCA nanosuspension was evaluated at room temperature (25 °C) and 4 °C at 7, 14, 30, and 60 days. Samples were tested in triplicate and stored in tightly closed amber glass vials.

In vitro physical stability was evaluated by means of particle size, size distribution, and zeta potential.

UDCA stability was evaluated by HPLC-UV [41]. In accordance with USP [45], UDCA content should be not less than 90% and not more than 110% of the labeled amount per unit of weight or volume. The initial concentration of UDCA was set as 100% and the remaining UDCA in the samples at each storage time was expressed as a percentage of the initial concentration.

To prepare the UDCA standard solution, 25 mg was accurately weighed into a 50 mL volumetric flask and diluted with methanol, and then further diluted (1/2) with mobile phase (ACN:H_3_PO_4_ pH 3.0; 0.15 mM) (48:52). On the other hand, each lyophilizate (corresponding to 5 mL of UDCA nanosuspension) was accurately weighed (~0.2 g) in a 5 mL volumetric flask and diluted with methanol. All samples were centrifuged at 14,000 rpm for five minutes to separate the insoluble components. Finally, a 1 mL aliquot was diluted to 5 mL with mobile phase (ACN:H_3_PO_4_ pH 3.0; 0.15 mM) (48:52), 100 µL of each sample were injected in triplicate into the HPLC-UV system, and detection was performed at 200 nm.

### 2.8. UDCA Oral Pharmacokinetics

Following oral administration of a single dose of UDCA to male Wistar rats (250–280 g), plasma drug concentration versus time profiles were plotted, in accordance with the published guideline for care and use of laboratory animals (NIH Publication N85-23, 1985, revise 1996). The animals were divided in two groups (*n* = 6) and then the oral pharmacokinetics of the freeze-dried UDCA nanosuspension (previously reconstituted in ultrapure water) were compared to those of a commercial UDCA tablet dispersed in ultrapure water. Formulations were orally administered by gavage employing a single UDCA dose of 7.5 mg/kg/day. Animals were maintained in a 12 h light/dark cycle at a 22 ± 2 °C environment. Standard rodent chow and water were provided ad libitum. After a minimum of 5 days of acclimatization to the new environment, they were fasted for 6 h before the oral administration of each formulation. Subsequently, blood aliquots (50 µL) were collected from the tail vein at 1.0; 2.0; 3.0; 4.0; 5.0; 8.0; 10; 12; and 24 h. Plasma samples were obtained by centrifuging blood samples at 10.000 rpm for 10 min. Supernatants were collected and deproteinized with cold acetonitrile and then SPE was performed through a C18 column (Phenomenex Strata C18-E, 500 mg/3 mL), following our previous work [46]. Plasma UDCA concentration was determined by a validated HPLC-MS/MS method previously reported [47].

#### Pharmacokinetic Parameters

Relative bioavailability was determined by plotting the natural logarithm of the plasma UDCA concentration as a function of time profiles, subsequently analyzed by a non-compartmental study (TOPFIT 2.0 software, Dr Karl Thomae Gmbh, Schering AG, Berlin, Germany). Peak plasma concentrations (C_max_); time to C_max_ (T_max_); the area under the curve from administration (t0) to 24 h (t24) (AUC0–24 h); and the IAUC_0–24h_ representing the increase in the area above baseline after UDCA ingestion and the ΔC_max_ (the maximal UDCA plasma concentration corrected by the basal value) were the estimated pharmacokinetic parameters.

The area under the curve was evaluated using the linear trapezoidal method. Results were expressed as the arithmetic mean of replicates ± SEM. Graphs and statistical analyzes were determined using the GraphPad Prism software (GraphPad 5.0, San Diego, CA, USA), normality through the Shapiro–Wilk test, differences between groups using Student’s paired test, and significance levels were established at *p* < 0.05.

## 3. Results and Discussion

### 3.1. Preparation of UDCA Nanosuspension

#### 3.1.1. Experimental Design

Due to the instability that nanosuspensions can present, such as particle aggregation due to their high surface energy, sedimentation, or crystalline growth, stabilizers are added to the formulation. They are adsorbed onto the particle surface and generate an electrostatic and/or steric repulsion, preventing such instability processes. Different types of stabilizers are used, which are classified as polymers such as HPMC, HEC, PVA, PVP, or TPGS; nonionic surfactants such as Tetronic, Pluronic, or Tween 80; and ionic surfactants such as SDS and soy lecithin, among others [3,48]. They are widely used in the development of nanosuspensions. Mishra B. et al. [21] and Luo Y et al. [49] used HPMC as a stabilizer, obtaining naproxen and simvastatin nanosuspensions, respectively, with adequate particle sizes. On the other hand, Hong et al. [48] designed myricetin nanosuspensions with different stabilizers, including TPGS, obtaining stable nanosuspensions with increased solubility, dissolution rate, and improved oral bioavailability. Rajamani et al. [50] developed naringenin nanosuspensions using TPGS as a costabilizer, which exhibited dose-dependent in vitro antitumor activity. On the other hand, Nakarani et al. [51], optimized itraconazole nanosuspensions, stabilized with poloxamer 407, which were chemically stable with a high API content and higher release when compared to a commercial formula.

A preliminary study with different stabilizers showed that UDCA nanosuspensions prepared with HPMC had the best combination of particle size (660 ± 6.45 nm) and narrow size distribution (Table 2). HPMC adsorbs by covering the hydrophobic surface of the API through hydrogen bonds and these interactions inhibit or slow crystal growth [52]. The other two stabilizers tested were discarded since, in the case of TETRONIC, an adequate particle size was observed but with a very high PDI, leading to a polydisperse unstable colloidal system. As for the TPGS, although it had a narrow PDI, the particle size was very large.

**Figure 1:**
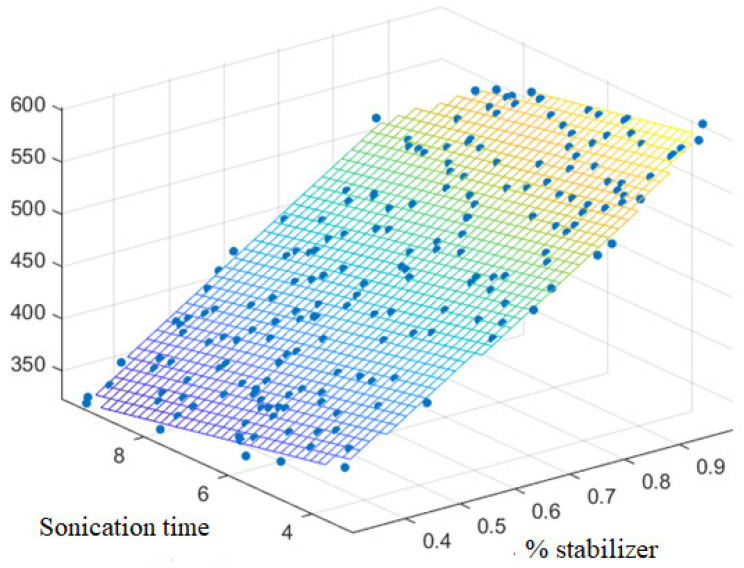
Response surface plot for the effect of stabilizer percentage ratio and sonication time on particle size.

A BBD of three factors and three levels 3^3^ was optimized. Table 3 shows the levels of each factor (−1, 0, and +1) with their corresponding real values, and the particle size as responses to study the influence of formulation and processing factors after performing 15 experiments. Among the proposed mathematical models, the linear model A is the one that best fits, the terms Y = β0 + β1 *X*_1_ + β1 *X*_3_ being significant; that is, the variables *X*_1_ and *X*_3_ (stabilizer percentage and sonication time, respectively). A polyplane was observed (Figure 1). By running the Matlab program and evaluating the response surface plot, the lowest particle size values were obtained at higher sonication times and lower HPMC percentage. Because of this, the conditions listed in Table 4 were selected. In the evaluated range, the model did not consider amplitude as a significant variable. After a preliminary study with different tests and amplitudes (30 W, 50 W, and 100 W), 50 W was selected as the most suitable operating amplitude since there were no significant differences in the particle size.

#### 3.1.2. Lyophilization of UDCA Nanosuspension

Freeze-drying or lyophilization represents a useful tool to improve the medium- and long-term stability of unstable liquid pharmaceutical formulations. This process involves sample freezing and ice sublimation (primary drying) along with desorption of unfrozen water (secondary drying) [53]. In recent years, lyophilization of biopharmaceuticals (recombinant proteins, antibodies, proteins, and vaccines) has attracted great attention in order to develop solid biopharmaceuticals with improved shelf life [54]. Furthermore, this process has also been extensively evaluated to stabilize different nano-sized formulations such as nanosuspensions [55,56,57], liposomes [58], lipid and polymeric nanoparticles [59], polymeric micelles [60,61], and nanocapsules [62].

**Figure 2:**
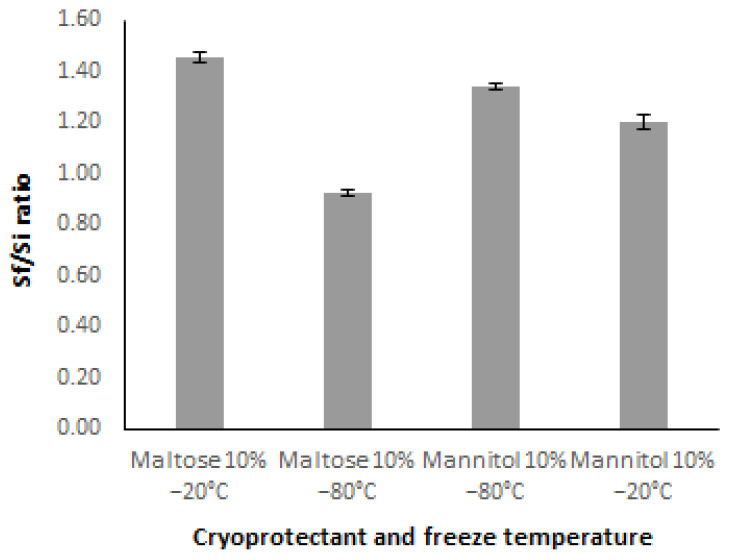
Sf/Si ratio shown by different cryoprotectants and temperatures in freeze–thaw studies.

In this framework, we aimed to obtain a freeze-dried powder from our UDCA nanosuspensions in order to enhance their physical stability over 60 days. Firstly, nanosuspensions were lyophilized and re-suspended in ultrapure water. However, they exhibited macro-sized aggregates, and their particle size was >6 μm, regardless of the freezing temperature. Lyophilization of nano-sized formulations represents a technological challenge. It is well known that the colloidal stability of the nanoformulations could be negatively influenced by both the freezing (crystal formation, phase separation) and the drying step (dehydration), leading to particle aggregation [53,63]. To overcome these drawbacks, the addition of cryo/lyoprotectants such as saccharides, polyols, and polymers are usually employed for the successful preparation of redispersible freeze-dried powders, taking into account the lyoprotectant concentration and the formulation properties [63]. Previously, Ma Y-Q et al. [31] evaluated the effect of solidification processes on the redispersibility of UDCA nanocrystals grown by HPH during lyophilization, employing different concentrations of stabilizers and cryoprotectants. They concluded that UDCA nanocrystals were subjected to agglomeration during solidification and the degree of agglomeration varied with the type and amount of stabilizers tested and with different solidification conditions. Hence, we employed two additives (maltose and mannitol) commonly used as cryo/lyoprotectants in nanoformulations [59,60]. Both additives were tested at 10% at two different freezing rates (−20 and −80 °C). Figure 2 shows a comparison of the Sf/Si values obtained for our UDCA nanosuspensions.

On one hand, those samples cryoprotected with mannitol demonstrated an increment of the particle size after lyophilization, regardless of the freezing temperature. For instance, the Sf/Si values were 1.21 and 1.34 for the nanosuspensions frozen at −20 and −80 °C, respectively. Moreover, samples exhibited different degrees of turbidity to the naked eye. On the other hand, a different behavior was observed after maltose incorporation into the UDCA nanosuspensions. Only those samples frozen at −80 °C demonstrated adequate Sf/Si values (0.93), while nanosuspensions frozen at −20 °C showed Sf/Si values over 1 ± 0.3 (Figure 2). Additionally, samples frozen at −80 °C remained translucent to the naked eye after they were re-suspended in ultrapure water. These results could be related to the lyoprotectant state after freeze-drying. Mannitol is a commonly lyoprotectant additive employed as a bulking agent for freeze-dried products, while saccharides promote the formation of a vitreous/glassy matrix. The former tends to crystallize adding extra mechanical stress to the nano-sized dispersion during the freeze-drying process. In contrast, maltose provides a vitreous matrix where the nano-sized dispersions are included and consequently protected from the mechanical stress [60,64]. Similar results were observed for sildenafil-citrate-loaded liposomes lyophilized with mannitol, lactose, sucrose, and trehalose [58]. Furthermore, higher freezing rates involve the formation of small ice crystals which could improve the stability of the nanoformulations [65,66]. In this framework, lower size increments were observed for those cryoprotected samples with maltose at a freezing temperature of −80 °C instead of −20 °C (Figure 2).

On the basis of the lowest Sf/Si and the appearance of the resuspended nanosuspensions, 10% maltose, and −80 °C were selected as the cryoprotectant and freezing temperature for the lyophilization of UDCA nanosuspensions.

### 3.2. Residual Solvent

Residual solvent content in the finished product must be evaluated and the permitted levels should be supported by safety data [67].

In nanosuspension formulations, acetone was used as a solvent for UDCA solubilization. According to the International Conference on Harmonisation (ICH), acetone belongs to class 3, with limits of 50 mg per day or less (corresponding to 5000 ppm), which can be considered less toxic and harmful to human health [67].

A good correlation was obtained with r^2^ = 0.9999 according to international guidelines. The LOD and LOQ results for the flame detector (FID) were 0.8 × 10^−9^ ppb and 2.5 × 10^−9^ ppb, respectively, and the LOD and LOQ results for the mass detector (MS) were 2.5 × 10^−12^ ppt and 2.5 × 10^−9^ ppb, respectively. In the samples analyzed by FULL SCAN or in SIM mode, no acetone was observed.

### 3.3. Characterization of Nanosuspensions

#### 3.3.1. Particle Size, Distribution, and Zeta Potential

Particle size of the lyophilized nanosuspension was 352.4 ± 3.90, with a polydispersity index of 0.11 ± 0.001 and a zeta potential of −4.30 mV ± 0.20. A suitable particle size was obtained. The nanosuspension was found to be monodisperse (PDI < 0.5), indicating a relatively narrow size distribution. UDCA nanosuspensions containing steric stabilizers may have low zeta potential values due to their adsorption. Because of this, they are stable as they respond to steric stabilization [68].

#### 3.3.2. SEM

**Figure 3:**
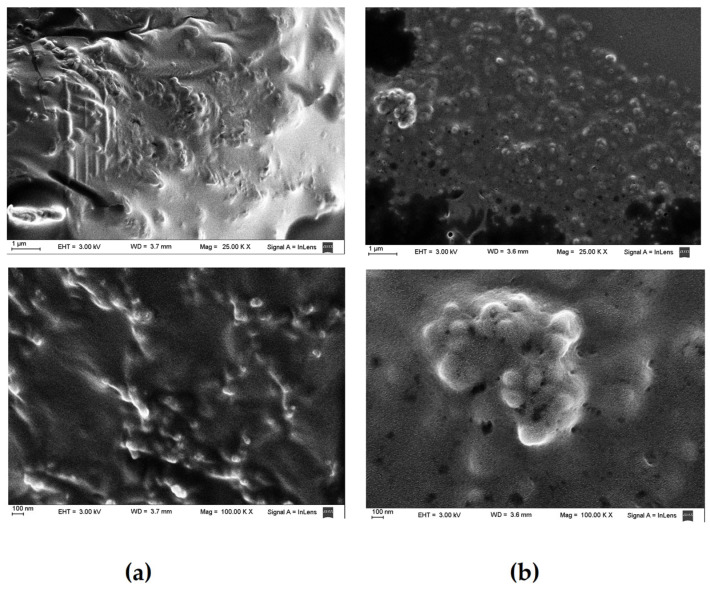
SEM (**a**): Freeze-dried UDCA nanosuspension; (**b**): reconstituted UDCA nanosuspension.

An inadequate lyophilization procedure could induce particle destabilization, leading to irreversible aggregation [39]. Due to this, the morphology of the lyophilized UDCA nanosuspension and the reconstituted nanosuspension were evaluated with SEM. As shown in Figure 3, the particles are covered by a “mantle” corresponding to the cryoprotectant (maltose). After freeze-drying the HPMC-stabilized nanosuspension, the particles formed flocs and exhibited slight aggregation, but no significant aggregation could be observed in the reconstituted nanosuspension. Therefore, a slight aggregation after lyophilization did not affect the redispersibility of the UDCA particles. Due to this, the lyophilized nanosuspension powder exhibited good redispersibility.

### 3.4. Dissolution Test

After 30 min, an amount equivalent to 86% of the UDCA content was determined in the dissolution medium. Therefore, it met the USP specification for dissolution testing.

### 3.5. Physicochemical Stability

#### 3.5.1. HPLC-UV Methods

The UDCA content was determined by HPLC-UV according to a previous work [41]. In this sense, the official USP UDCA monograph describes a liquid chromatography (LC) method coupled to a differential refractive index detector due to the low absorptivity of bile acids. In recent years, several analytical methods have been developed for UDCA determination and quantification, such as HPLC-MS/MS applied to the determination of UDCA in biological samples with high sensitivity and specificity [69,70], gas chromatography with mass detection (GC-MS), and capillary electrophoresis with UV detection (CE-UV). However, GC-MS requires deconjugation and derivatization of bile acids, and CE-UV, although adequate for the determination of UDCA in pharmaceutical formulations, requires large amounts of a sample [71,72]. Our research group developed and validated a simple, fast, specific, exact, and robust method with HPLC-UV for the determination and quantification of UDCA in raw materials and liquid formulations [41].

After a literature search, it was found that most of the HPLC methods for the determination of UDCA in different matrices used C18 columns. Therefore, in the analytical method development, a RP-C18 column was selected (Thermo (150 mm, 4.6 mm, 5 µm)), but a poor peak shape was obtained. For this reason, a Symmetry column was used instead, and an excellent peak shape was obtained, achieving greater sensitivity and specificity. The official USP UDCA monograph describes an acidic mobile phase. On the other hand, most of the HPLC methods for UDCA analysis in different matrices use buffers at acidic pH values as mobile phases. Due to this, we validated the method using an acid mobile phase (acetonitrile: phosphoric acid (pH 3.0; 0.15 mM) (48:52)), obtaining an optimal resolution between UDCA and excipients [41,45,73].

#### 3.5.2. Stability

Table 5 presents the particle size and distribution, and zeta potential in the lyophilized nanosuspensions at 25 °C and 4 °C, respectively, for 60 days. Particle size values at both temperatures for 60 days were similar with no significant variations. As for PDI, the nanosuspensions turned out to be monodisperse (PDI < 0.5) throughout the stability study. HPMC has sufficient affinity for the particle surface and possesses a high enough diffusion rate to adequately cover the generated surface after ultrasound application, which provides sufficient steric repulsion between the particles to contribute to the stability during storage [22]. In turn, the zeta potential values also remained similar without significant variations. In general, a zeta potential of at least −20 mV is sufficient to stabilize the nanosuspension system [48]. Although the nanosuspensions remained stable for 60 days, a higher deviation was observed at 25 °C in terms of particle size.

Table 6 shows the UDCA content in the lyophilized nanosuspension at 25 °C and 4 °C, respectively, for 60 days. The UDCA content at both temperatures complies with the USP specification since it is between 90% and 110% with an RSD of less than 2%. In terms of content, the lyophilized nanosuspension at 4 °C presented higher variability.

### 3.6. UDCA Oral Pharmacokinetics

A preliminary study of the relative oral bioavailability of UDCA in male Wistar rats was carried out where the lyophilized nanosuspension was compared with the commercial tablet, both formulations being resuspended in water prior to administration.

**Figure 4:**
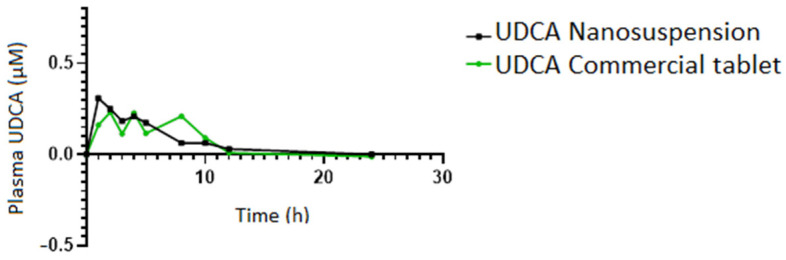
UDCA plasma concentration–time curve (μM) for 24 h in rats after a single-dose oral administration of UDCA nanosuspension and UDCA commercial tablet.

Figure 4 shows the UDCA plasma concentration curve (µM) in rats after administration of a single dose of UDCA and a non-compartmental analysis. The pharmacokinetic parameters in the nanosuspension vs. the commercial tablet were T_max_ (1.0 ± 0.9 h vs. 2.0 ± 0.8 h, respectively), C_max_ (0.558 ± 0.118 vs. 0.366 ± 0.113 µM, respectively), ΔC_max_ (0.309 ± 0.099 vs. 0.232 ± 0.056, respectively), AUC (4.326 ± 0.471 vs. 2.188 ± 0.353 µg/mL.h, respectively, *p* < 0.02), and IAUC_0–24h_ (2.261 ± 0.187 µg/mL.h vs. 1.924 ± 0.440 µg/mL.h, respectively). Although a potential significant difference may be observed in the increase in the AUC for the nanosuspension, the IAUC (which considers the variable of the basal levels) shows only a trend. It is possible that the great variability of UDCA basal levels in different animals overshadows a potential significant difference. However, given the endogenous nature of UDCA, the IAUC is a more appropriate parameter than the AUC to draw any explanation from. This is why we conclude that the observed data reveal that the nanosuspension and the commercial tablet had similar relative bioavailability without significant statistical differences.

On the other hand, a more erratic behavior is observed in UDCA plasma levels with the commercial tablet compared to the nanosuspension.

## 4. Conclusions

The application of the precipitation–ultrasonication technique turned out to be optimal for the development of UDCA nanosuspensions, obtaining a small and uniform particle size. The analysis of the BBD revealed that the linear model was the best fit since it showed that the stabilizer percentage and sonication time had a significant effect on particle size. The lyophilization process rendered UDCA nanosuspensions with adequate particle size, and monodispersed, which indicates a relatively narrow size distribution. In turn, the selected cryoprotectant allowed an adequate redispersibility of the UDCA particles. The UDCA nanosuspension presented adequate physical stability for 60 days at 25 °C and 4 °C, since the UDCA content ranged between 90–110%, complying with USP specifications. Although the in vivo studies demonstrated that the relative UDCA oral bioavailability was similar, without significant differences, when the lyophilized nanosuspension and the commercial tablet were administered, the former exhibited a less erratic plasma profile in the observed period compared to the tablet.

For all of the above, the development of UDCA nanosuspensions is promising, since it is easy to prepare and requires a minimum amount of excipients, which leads to less toxicity and side effects. Moreover, the developed formulation remained stable throughout the study. Even though in vivo studies of UDCA nanosuspensions are necessary to evaluate their efficacy and safety, our findings show that adequate UDCA oral absorption and bioavailability were achieved.

## Figures and Tables

**Table 1:** Variables and respective levels in the BBD for UDCA nanosuspension preparation.

		Levels		
Independent Variables		Low (−1)	Medium (0)	High (+1)
*X*_1_	Stabilizer (%)	0.3	0.5	1.0
*X*_2_	Amplitude (W)	30	50	100
*X*_3_	Sonication time (min)	3	5	10
Dependent variable			Constraints	
*Y*_1_	Particle size (nm)		Minimize	

**Table 2:** Particle size and distribution (PDI) in the UDCA nanosuspensions with different stabilizers.

Stabilizer	Particle Size * (nm)	PDI *
HPMC E-15	660 ± 6.45	0.313 ± 0.034
TETRONIC 1107	489 ± 16.12	0.984 ± 0.022
TPGS	992 ± 12.87	0.145 ± 0.061

* Mean value (*n* = 3) ± SD (standard deviation).

**Table 3:** BBD variables and levels and UDCA nanosuspension particle size.

Formula	Levels			Response
	Stabilizer (%)	Amplitude (W)	Sonication Time (min)	Size (nm)
N1	0.5	100	3	399.0
N2	1.0	50	10	421.2
N3	1.0	100	5	522.3
N4	1.0	30	5	694.7
N5	0.5	30	10	398.1
N6	0.3	50	10	354.1
N7	0.3	100	5	333.4
N8	0.3	50	3	351.7
N9	0.3	30	5	351.1
N10	0.5	100	10	412.8
N11	0.5	30	3	417.4
N12	1.0	50	3	664.5
N13	0.5	50	5	411.2
N14	0.5	50	5	413.1
N15	0.5	50	5	403.8

**Table 4:** Optimized factors and levels used for UDCA nanosuspension.

Factors	Factor Levels
X_1_: Stabilizer (%)	HPMC 0.3%
X_2_: Amplitude (W)	50
X_3_: Sonication time (min)	5

**Table 5:** Physical stability of the lyophilized nanosuspension at 4 °C and 25 °C.

Lyophilized Nanosuspension						
TIME (Days)	25 °C			4 °C		
	Particle Size * (nm)	PDI *	Z Potential * (mV)	Particle Size * (nm)	PDI *	Z Potential * (mV)
0	522.0 ± 7.11	0.204 ± 0.021	−4.43 ± 0.21	459.6 ± 1.13	0.232 ± 0.011	−3.01 ± 0.03
7	507.4 ± 0.71	0.182 ± 0.020	−3.28 ± 0.21	466.2 ± 1.84	0.171 ± 0.004	−3.79 ± 0.08
14	497.4 ± 5.81	0.212 ± 0.001	−3.58 ± 0.16	491.4 ± 4.24	0.206 ± 0.004	−4.14 ± 0.12
30	525.5 ± 6.36	0.177 ± 0.022	−2.21 ± 0.15	487.7 ± 1.98	0.207 ± 0.004	−3.09 ± 0.08
60	458.6 ± 8.14	0.212 ± 0.010	−4.30 ± 0.17	426.0 ± 2.63	0.235 ± 0.016	−3.70 ± 0.23

* Mean value (*n* = 3) ± SD (standard deviation).

**Table 6:** Chemical stability of lyophilized nanosuspension at 4 °C and 25 °C. Determination of UDCA content.

Lyophilized Nanosuspension		
TIME (Days)	4 °C (%)	25 °C (%)
0	100.0 (1.98)	100.0 (1.95)
7	103.9 (1.03)	100.0 (1.98)
14	105.3 (1.07)	100.0 (0.30)
30	100.0 (0.57)	102.6 (2.00)
60	107.9 (0.63)	103.8 (0.50)

RSD values between brackets corresponding to *n* = 3.

## Data Availability

The data presented in this study are available on request from the corresponding author.
